# Dual Source Photon-Counting Computed Tomography—Part II: Clinical Overview of Neurovascular Applications

**DOI:** 10.3390/jcm12113626

**Published:** 2023-05-23

**Authors:** Filippo Cademartiri, Antonella Meloni, Laura Pistoia, Giulia Degiorgi, Alberto Clemente, Carmelo De Gori, Vincenzo Positano, Simona Celi, Sergio Berti, Michele Emdin, Daniele Panetta, Luca Menichetti, Bruna Punzo, Carlo Cavaliere, Eduardo Bossone, Luca Saba, Riccardo Cau, Ludovico La Grutta, Erica Maffei

**Affiliations:** 1 Department of Radiology, Fondazione Monasterio/CNR, 56124 Pisa, Italy; antonella.meloni@ftgm.it (A.M.); laura.pistoia@ftgm.it (L.P.); giuliadegiorgi995@gmail.com (G.D.); clemente@ftgm.it (A.C.); degoricarmelo87@gmail.com (C.D.G.); positano@ftgm.it (V.P.); emaffei@ftgm.it (E.M.); 2 Department of Bioengineering, Fondazione Monasterio/CNR, 56124 Pisa, Italy; 3 BioCardioLab, Department of Bioengineering, Fondazione Monasterio/CNR, 54100 Massa, Italy; simona.celi@ftgm.it; 4 Cardiology Unit, Ospedale del Cuore, Fondazione Monasterio/CNR, 54100 Massa, Italy; sergio.berti@ftgm.it; 5 Department of Cardiology, Fondazione Monasterio/CNR, 56124 Pisa, Italy; emdin@ftgm.it; 6 Institute of Clinical Physiology, National Council of Research, 56124 Pisa, Italy; daniele.panetta@ifc.cnr.it (D.P.); luca.menichetti@ifc.cnr.it (L.M.); 7 Department of Radiology, IRCCS SynLab-SDN, 80131 Naples, Italy; bpunzo@sdn-napoli.it (B.P.); carlo.cavaliere@synlab.it (C.C.); 8 Department of Cardiology, Ospedale Cardarelli, 80131 Naples, Italy; ebossone@hotmail.com; 9 Department of Radiology, University Hospital, 09042 Monserrato, Italy; lucasabamd@gmail.com (L.S.); riccardocau00@gmail.com (R.C.); 10 Department of Health Promotion, Mother and Child Care, Internal Medicine and Medical Specialties-ProMISE, Department of Radiology, University Hospital “P. Giaccone”, 90127 Palermo, Italy; lagruttaludovico@gmail.com

**Keywords:** photon-counting computed tomography, computed tomography angiography, neurovascular, photon-counting detector, energy integrating detector

## Abstract

Photon-counting detector (PCD) is a novel computed tomography detector technology (photon-counting computed tomography—PCCT) that presents many advantages in the neurovascular field, such as increased spatial resolution, reduced radiation exposure, and optimization of the use of contrast agents and material decomposition. In this overview of the existing literature on PCCT, we describe the physical principles, the advantages and the disadvantages of conventional energy integrating detectors and PCDs, and finally, we discuss the applications of the PCD, focusing specifically on its implementation in the neurovascular field.

## 1. Introduction

Computed tomography (CT) is currently one of the cornerstone imaging modalities in clinical use. It is available in standard and emergency settings almost worldwide, with application in the diagnosis of a wide array of conditions. It provides three-dimensional images of the linear attenuation coefficient distribution within a patient, accurately delineating organs and tissues [1].

The X-ray detector is a key part of a CT scanner, having a critical role in image quality and radiation dose. All modern commercial CT scanners utilize solid-state detectors and have a third generation rotate–rotate design [2]. In CT imaging, the classification of different types of tissues can be challenging because materials with different elemental compositions can be represented by the same or very similar CT numbers, for instance, calcified plaques or adjacent bone may be indistinguishable from iodine-containing blood.

In addition to the difficulty in differentiating and classifying tissue types, the accuracy with which material concentration can be measured is degraded by the presence of multiple tissue types. For example, when measuring the amount of iodine enhancement of a soft-tissue lesion, the measured mean CT number over the lesion reflects not only the enhancement due to iodine, but also the CT number of the underlying tissue. The reason for the forementioned difficulties derives from the fact that the measured CT number of a voxel is connected to its linear attenuation coefficient, which is in turn the result of the combination between the material composition, the photon energies interacting with the material, and the mass density of the material, and as such, is not unique for any given material.

A recent, notable development in the field of CT is the analysis of spectral information of the X-rays that have passed through the subject. A variety of systems, known under the term of dual-energy CT (DECT), allow spectral separation, either based on energy-integrating detectors (EIDs) or photon-counting detectors (PCDs). PCD technology is a novel approach to acquire multiple energy datasets, having various benefits over conventional EIDs [3,4].

We will briefly illustrate the physical principles of EIDs and PCDs as well as the advantages and disadvantages of each one, and finally, we will discuss the applications of PCDs, focusing specifically on their implementation in the neurovascular field.

## 2. Search Strategy

This is a narrative review, prepared according to the indications present in [5]. The article search was performed on PubMed, Scopus, and Google Scholar electronic databases between December 2022 and January 2023. We used the keywords “photon-counting computed tomography”, “PCCT”, “photon counting detector”, “photon counting X-ray detectors”, “photon counting CT”, and “spectral CT”. Only articles written in English were included. Additional records identified through the list of references or other sources were also included.

## 3. Conventional EID Technology

Standard CT detectors use a two-step indirect conversion technique. First, a scintillator converts the X-ray energy into visible light. Second, visible light is collected and converted into an electrical charge using a photodiode [6,7]. The output signal of the detector is proportional to the total energy deposited by all detected X-ray photons; thus, the name, energy-integrating detectors. Since the generated beam is polyenergetic and EIDs weight the measured signal according to the energy of the detected photon, lower energy photons contribute less than the higher energy photons in the output signal, resulting in increased noise and decreased contrast [8].

Moreover, as the spatial spread of visible light inside the scintillator is very strong, the individual pixels of the detector need to be separated by reflective material to keep the light inside the ceramic and to allow only the light to be emitted towards the photodiode. Therefore, the pixels cannot be made arbitrarily small since the area between the pixels does not take part in the conversion process and, therefore, is lost when measuring the photons [8].

Multienergy CT that uses conventional EIDs can be divided into source-based CT technologies (Dual-source CT, Rapid kVp Switching CT, Split-beam CT) and detector-based technologies (Dual layer CT) [6,9,10,11].

## 4. PCD Technology

In addition to the overall amount of X-ray intensity, the photon-counting detectors enable measurement of each incoming X-ray photon individually through a direct conversion technology using a fast semiconductor sensor with high stopping power, such as cadmium-telluride (CdTe) or cadmium-zinc-telluride (CZT). Other materials, such as silicon and gallium arsenide, have also been used.

The direct conversion principle makes it possible to downsize pixels and eliminate reflective septa, resulting in higher resolution. The process starts with an incident X-ray, which is absorbed in the semiconductor, causing the creation of a cloud of positive and negative charges (i.e., electron hole pairs), whose amount is a function of the energy of the photon. The positive and negative charges are pulled away from each other rapidly, and the moving electron charges induce an electrical signal in the respective pixel electrode that is registered through an electronic readout circuit [12].

So, each photon hitting the detector element generates an electrical pulse with a height proportional to the energy deposited by the photon. Then, the electronics system of the detector counts the number of pulses with heights that exceed a preset threshold level. The threshold is set at levels that are higher than the electronic noise level (thus eliminating it and diminishing radiation dose and artifacts with it) but lower than pulses generated by incoming photons. Furthermore, by comparing every pulse to several threshold levels, the detector can sort the incoming photons into sets of energy bins, depending on their energy [12,13,14].

## 5. Advantages and Disadvantages of PCDs

The PCCT presents many benefits for the diagnostic field.

Firstly, owing to the higher resolution, it improves the visualization of small structures in many diagnostic fields:

- in pulmonary imaging, it better displays bronchial walls, and the pathologic changes that can occur in normal parenchyma, for instance, ground-glass opacity and reticulations [15,16];
- in skeletal imaging, it can identify lytic lesions and pathologic fractures in myeloma, with a similar dose compared to low-dose CT [15], and it can allow for a superior anatomical delineation of the submillimeter structures of the sinus and temporal bone [17];
- in the renal system, it can characterize smaller renal stones (3 mm and less) than conventional CT [18].

Secondly, it improves iodine contrast at the same tube potential as compared to EID-CT, due to the correct counting of low-energy photons, which are, on the other hand, downweighed in conventional EIDs. Improvements in iodine signal with PCCT can also be used to reduce the amount of iodine used to achieve similar differences in image contrast for different diagnostic tasks in those patients who have renal disease. 

Thirdly, PCDs allow acquisition of simultaneous multienergy images at a single X-ray tube owing to the energy-discriminating ability. This enables material decomposition (MD), plaque removal, bone removal, and virtual monoenergetic images (VMIs), such as virtual noncontrast (VNC) imaging, virtual noncalcium imaging, and iodine images [8,15].

Fourthly, PCDs use the highest possible voltages (140 kVp) in order to have the largest photon spectrum. The technology based on 140 kVp allows to effectively reduce beam hardening artifacts without compromising the soft tissue–iodine contrast [19] and to reduce noise in the obese patient.

Lastly, PCCT can avoid sedation and produce images with high spatial resolution and contrast-to-noise ratio with increased dose efficiency, facilitating dose reduction in pediatric populations [15,20]. Compared to conventional EID-CT, a significant dose reduction for sinus imaging and for temporal bone imaging has been demonstrated in PCCT images acquired at high-voltages with an additional tin filter [17,21]. Indeed, PCDs have eliminated the need for dose-inefficient comb/grid filters for ultra-high resolution imaging. 

The advantages of PCD CT are, therefore, many and are summarized in Table 1. 

However, certain limitations intrinsic to this technology must be considered. PCDs cannot function properly with high count rates. In fact, high count rates can cause two photons being absorbed very close together in time and being incorrectly counted as a single photon with an energy equal to the sum of the energy of both photons. This effect, known as electronic pileup, may result in a reduction in the energy resolution and affect image quality. This is one of the reasons why there is growing interest in having fast readout electronics and small detector pixels so as to decrease the count rate per pixel. However, if, on the one hand, reducing the pixel size can reduce the pileup effect, on the other hand, it can lead to an increase in a phenomenon called charge sharing, consisting of the electron charge cloud caused by photon absorption in the detector being shared between two nearby pixels, also creating distortions in the spectral response [7,22,23,24]. Anyway, it has been demonstrated that, despite the use of high fluxes (up to 550 mA at 140 kV) and wide water phantoms, the prototype PCD system suffered from negligible pileup effects [25].

## 6. Clinical Applications in the Neurovascular Field

Table 2 summarizes the studies regarding PCCT in neurovascular imaging.

### 6.1. Iodine Maps

The angiography of head and neck is a pivotal step in the diagnosis of acute cerebral conditions, such as ischemic stroke and intracranial hemorrhage. In this regard, the iodine map is useful for improving the visualization of extracranial and intracranial vessels [39].

**Figure 1:**
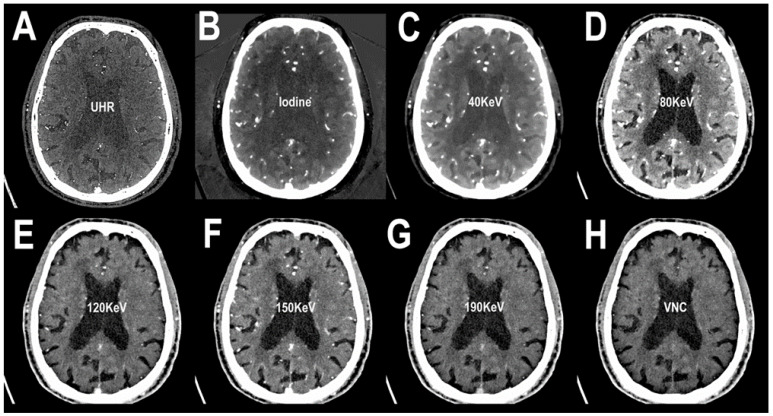
PCCT angiography of the head: monochromatic imaging. The figure shows an axial slice image from a brain scan performed in the arterial phase after intravenous contrast material administration. In (**A**), the ultra-high resolution (matrix 1024 × 1024; slice thickness/increment 0.2/0.1 mm; voxel 100 microns; convolution kernel B60f; radiation dose comparable to equivalent CT angiography of the head with comparable Dual-source CT of the 3rd generation) is visualized. Since the acquired images are enriched with the entire KeV spectrum, in (**B**), the same slice with an iodine spectrum is visible. From (**C**–**G**), the same slice is showed in different KeV settings, starting from 40 KeV up to 190 KeV. While the image can be reconstructed with the iodine spectrum as in (**B**), it is also possible to reconstruct and subtract the iodine spectrum (i.e., the contrast enhancement determined by iodinated contrast material) creating a virtual noncontrast image (VNC) (**H**). From a single PCCT acquisition, it is possible to derive several multiparametric information.

**Figure 2:**
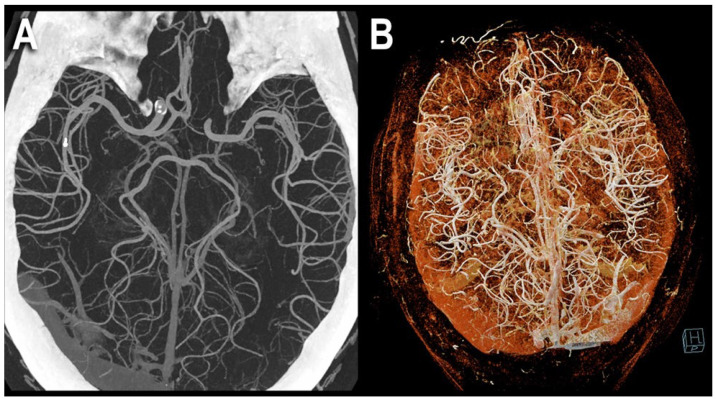
PCCT angiography of the head: 3D cinematic volume rendering display. The figure shows a PCCT angiography of the brain with ultra-high resolution of 100 microns (source dataset; matrix 1024 × 1024; slice thickness/increment 0.2/0.1 mm; convolution kernel B60f; radiation dose comparable to equivalent CT angiography of the head with comparable Dual-source CT of the 3rd generation) using volumetric 3D Maximum Intensity Projection (MIP) (**A**) and cinematic volume rendering (**B**). To be noted is the massive increase in the density of the small arterial vessels displayed.

**Figure 3:**
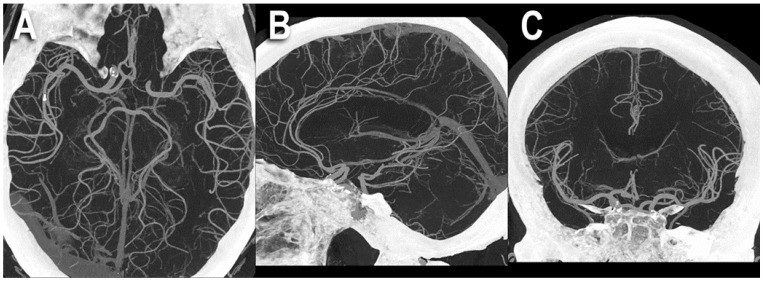
PCCT angiography of the head: MIP volumetric display. The figure shows a PCCT angiography of the brain with ultra-high resolution of 100 microns (source dataset; matrix 1024 × 1024; slice thickness/increment 0.2/0.1 mm; convolution kernel B60f; radiation dose comparable to equivalent CT angiography of the head with comparable Dual-source CT of the 3rd generation) using thick-slab 3D Maximum Intensity Projection (MIP) in the three main planes: axial (**A**), sagittal (**B**), and coronal (**C**). To be noted is the massive increase in the density of the small arterial vessels displayed and the length of the segments visualized; for arteria cerebralis posterior and media in (**A**), for arteria cerebralis anterior in (**B**), and for arteria cerebralis media in (**C**).

Examples of PCCT angiography of the head are shown in Figure 1, Figure 2 and Figure 3.

An in vivo human study of major vessels of head and neck by Symons et al. [19] compared PCD technology and EID technology, finding that the first one performed better for image noise (9.1% lower than EID) and image artifacts.

In a study by Riederer et al. [26], the role PCCT can play in differentiating blood from hemorrhagic transformation of infarction from iodine leakage in comparison to conventional CT was researched in vitro and ex vivo on a bovine brain model. Hyperdense areas are, in fact, seen frequently after neurovascular procedures and the differentiation between the two entities is relevant since it has been demonstrated that contrast extravasation is a predictor of poor clinical outcomes in patients undergoing endovascular therapy for acute ischemic stroke [40]. Riederer et al. showed that in conventional CT imaging, the HU density of blood and iodine were similar both in vitro and in the bovine ex vivo model, whereas the iodine maps of PCCT enabled clear differentiation between the two and precise quantification of iodine [26]. 

A phantom study on the same topic by Van Hedent demonstrated that spectral PCCT could accurately differentiate blood from iodinated contrast (*p* < 0.01) [27].

These findings are in line with previous studies that compared DECT and conventional CT on the same topic: for instance, the works by Tijssen et al. [41], Mangesius et al. [42], and Zaouak et al. [43].

PCCT can also be used to assess intracerebral hemorrhage of unknown (ICH) origin, differentiating between tumor bleeding and pure intracerebral hemorrhage, for which DECT has already been shown to be effective [44].

Michael et al. recently evaluated whether monoenergetic reconstructions (MERs) or polyenergetic reconstructions (PERs) offered the best image quality in the angiography of head and neck through qualitative assessment and quantitative assessment by signal noise, signal-to-noise ratio (SNR), and contrast-to-noise ratio (CNR) [28]. These results differed from those of previously published studies, the key finding being that PERs at this current time perform better than MERs.

### 6.2. Artifact Reduction

Metal artifacts pose a problem in CT, being detrimental to its diagnostic value since the dark and bright streaks can hinder detection of a lesion in the underlying tissue. For instance, neurocoils used in the treatment of cerebral aneurysms may prevent periprocedural hemorrhage or ischemia from being identified, as well as the evaluation of aneurysm reperfusion. Through its ability to sort the incoming photons into different energy bins, PCCT represents a solution to this issue. The higher energy photons, in fact, suffer less from the beam hardening effect, which make up for a large part of metal artifact; therefore, the highest bin image is less susceptible to metal artifacts than conventional CT image [45].

A recent in vitro study, published in 2023 by Schmitt et al. [29], compared coil-related artifacts in PCCT and the conventional energy-integrating detector CT (EID-CT) using an equivalent standard brain imaging protocol before and after metal artifact reduction (MAR). This investigation showed a higher degree of coil-related artifacts in the total-energy PCCT and low-energy PCCT as opposed to the EID-CT and no significant difference in coil-related artifacts between conventional CT and high-energy bin PCCT without MAR, whereas total-energy PCCT combined with MAR showed diminished metal artifacts compared with EID-CT.

In another investigation by Do et al. [30], the influence that PCCT tube potential, energy thresholding, and acquisition mode (Macro versus Chess) have on metal artifact and image noise was evaluated, concluding that metal artifacts can be reduced by using PCCT in combination with high-energy thresholds instead of energy-integrating detectors. 

Furthermore, differences between acquisition modes were observed for metal artifact reduction in different target structures. Macro-high-energy Threshold Images (HTI), where the same threshold is applied for all pixels, effectively grouping all subpixels together, showed better results for cortical bone. Chess-HTI, in which the subpixels are assigned in an alternating manner to two different threshold settings, thus allowing four energy thresholds, seemed to produce fewer artifacts for bone marrow but at further increased image noise.

Another source of metal artifact is represented by dental implants or dental fillings, hampering the correct localization of tumors in the surroundings, which in turn is vital to plan cancer treatment. Lee et al. addressed this problem and explored the potential of PCCT in the individuation of oral tumors located under metal artifact, using an ATOM phantom with metal insertions to verify the effect of the proposed metal artifact reduction [31]. Three energy thresholds (30 keV, 50 keV, and 65 keV) were used to group data into three bins (low-energy, middle-energy, and high-energy, respectively) in the PCCT. Normalized metal artifact reduction (NMAR) was performed for each energy bin image, followed by an image-based MD and VMI reconstruction. Image quality was analyzed quantitatively by contrast-to-noise ratio (CNR) measurements. The results included good iodine estimation of the true iodine content, better contrast between iodine and the surrounding material in bin 1 image, and fewer metal artifacts in bin 3 than the bin 1 image because of the higher photon energy. The result of quantitative assessment demonstrated that the 40 keV VMI (CNR = 20.6 ± 1.2) with NMAR and MD remarkably increased the contrast of the iodine-enhanced region compared with that of the conventional images (CNR = 10.4 ± 0.5) having 30 to 140 keV energy levels. Therefore, this work showed the potential PCCT has to maximize the contrast of the target tissue while reducing metal artifacts.

### 6.3. Increased Resolution

One of the challenges with brain CT is the differentiation between grey matter (GM) and white matter (WM), whose loss is a sign to look for when evaluating the presence of early ischemic brain damage or other pathological conditions. Moreover, the degradation of the image caused by the beam artifacts coming from the bone structures can further reduce the GM–WM differentiation [46,47].

Pourmorteza et al. researched ex vivo and in vivo whether PCCT offered better image quality in comparison with conventional CT. Ex vivo, PCD images showed 8.5% ± 4.8% less image noise than EID images, whereas, in vivo, an improvement of 12.8–20.6% for image noise, 19.0–20.0% for SNR, 15.7% for GM–WM, and 33.3% for GM–WM CNR was registered [32]. These findings suggest the high potential of PCCT to improve image quality in the brain or, alternatively, might allow a reduction in the radiation dose (approximately 40%) at similar quality levels of current brain CT.

### 6.4. Tissue Analysis

A potential advantage of PCCT is the opportunity to better assess the composition of the tissue by using a quantitative approach. Owing to the PCCT physical principles, it is possible to create quantitative maps of the distribution of some specific atomic elements within a tissue. 

A recently published paper by Dahal et al. demonstrated the effectiveness of PCCT in quantifying (in comparison with histopathological measurements) fibrous cap (FC) thickness, FC area, and lipid-rich necrotic core (LRNC) area in excised atherosclerotic carotids, due to the combined effect of high spatial and energy resolution of PCCT [33]. These results are in line with those that already emerged from works on the coronary arteries [48,49].

**Figure 4:**
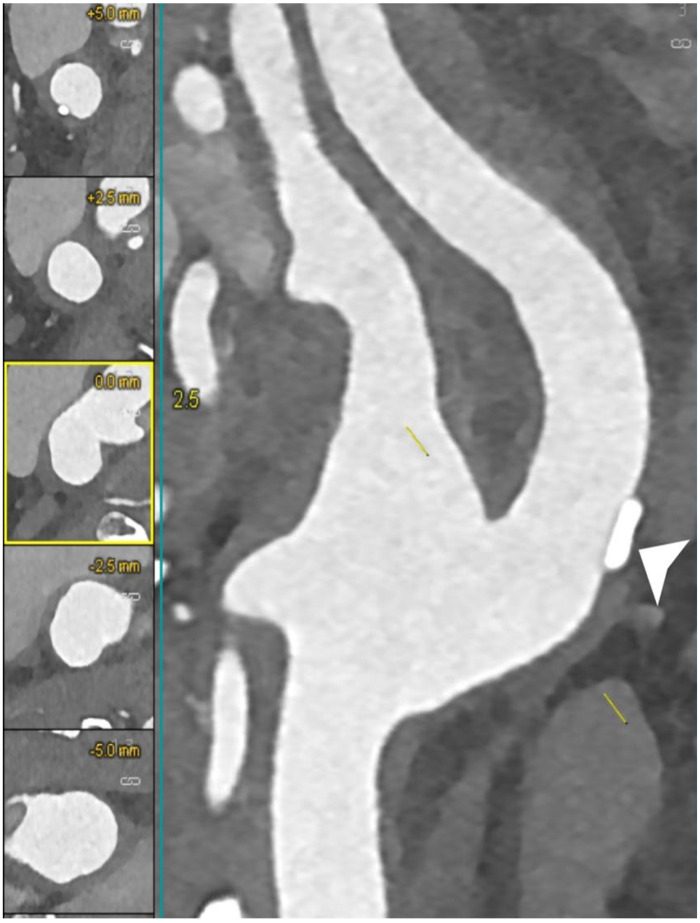
PCCT angiography of carotid artery bifurcation: mild calcified atherosclerosis. The PCCT angiography shows an ultra-high-definition (source dataset; matrix 1024 × 1024; slice thickness/increment 0.2/0.1 mm; voxel 100 microns; convolution kernel B60f; radiation dose comparable to equivalent CT angiography of the carotids with comparable Dual-source CT of the 3rd generation) carotid bifurcation with arterial wall thickening and mildly focal calcified plaque at the level of the proximal internal carotid artery (arrowhead).

**Figure 5:**
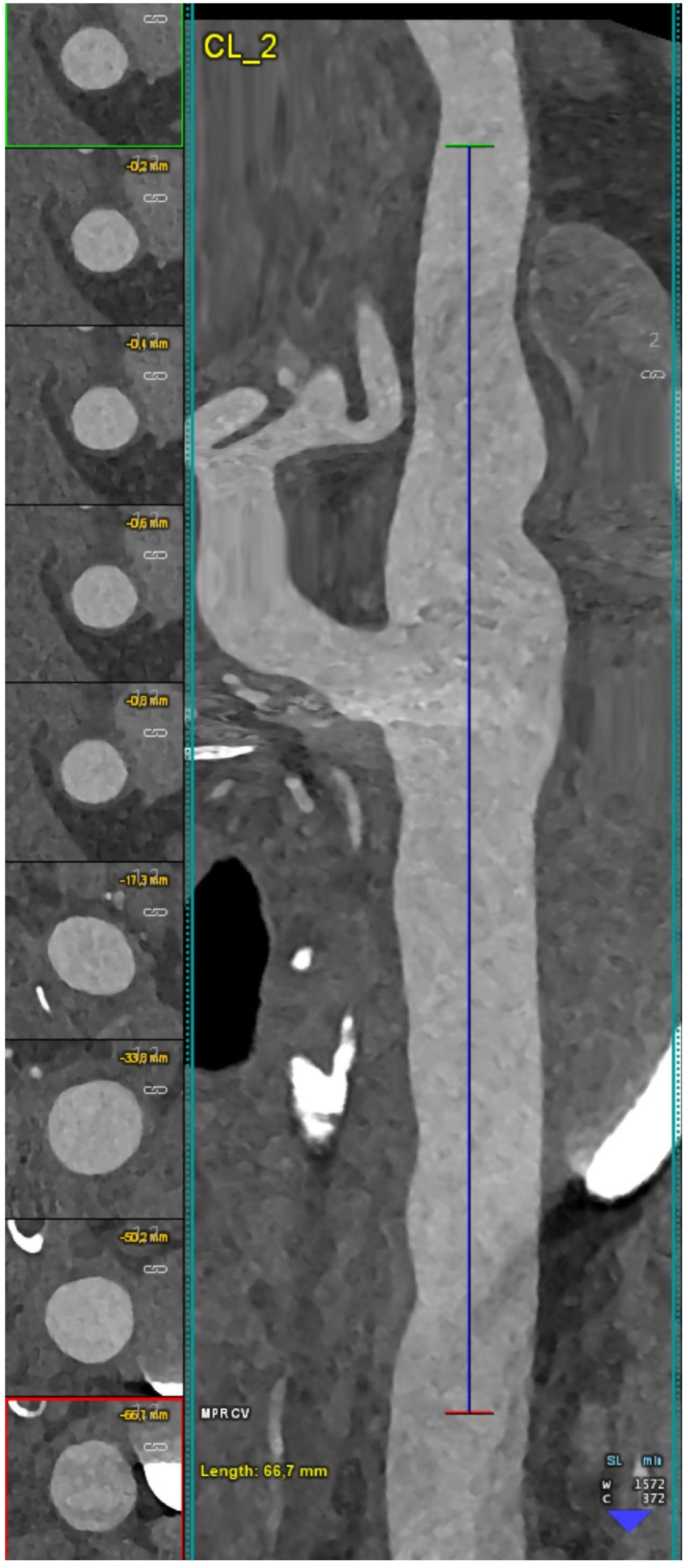
PCCT angiography of carotid artery bifurcation: mild non calcified atherosclerosis. The PCCT angiography shows a longitudinally stretched curved multiplanar reformat of an ultra-high-definition (source dataset; matrix 1024 × 1024; slice thickness/increment 0.2/0.1 mm; voxel 100 microns; convolution kernel B60f; radiation dose comparable to equivalent CT angiography of the carotids with comparable Dual-source CT of the 3rd generation) carotid bifurcation with mild diffuse arterial wall noncalcified atherosclerosis.

**Figure 6:**
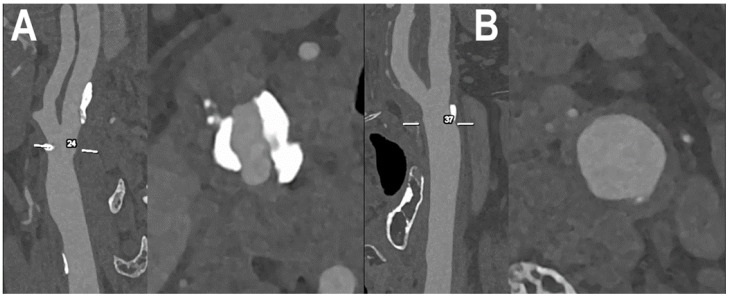
PCCT angiography of carotid artery bifurcations: intermediate mixed and calcified atherosclerosis. The PCCT angiography shows 2 ultra-high-definition (source dataset; matrix 1024 × 1024; slice thickness/increment 0.2/0.1 mm; voxel 100 microns; convolution kernel B60f; radiation dose comparable to equivalent CT angiography of the carotids with comparable Dual-source CT of the 3rd generation) carotid bifurcations with severely calcified atherosclerotic plaque ((**A**); longitudinal and axial view) and intermediate/moderate mixed/calcified plaque ((**B**); longitudinal and axial view). To be noted is how the bulky and massive calcification of the plaque in (**A**) is totally distributed within the arterial wall and not affecting the visualization and quantification of eventual lumen stenosis.

**Figure 7:**
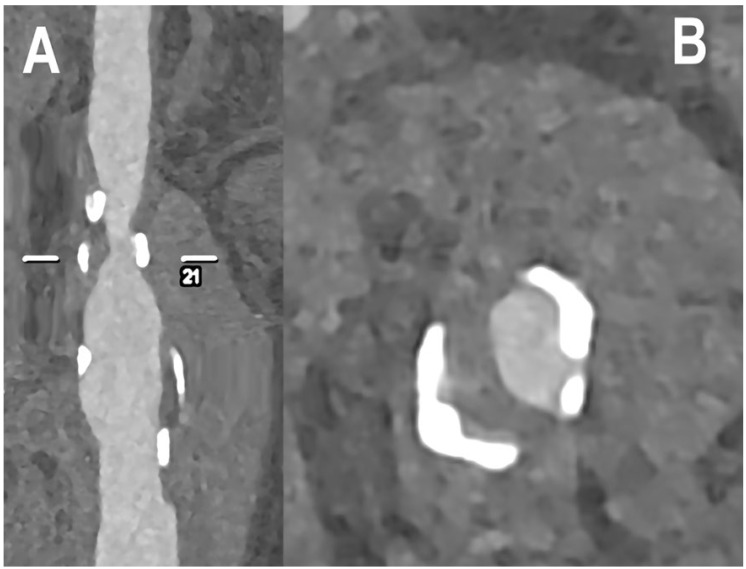
PCCT angiography of carotid artery bifurcation: severe mixed atherosclerosis. The PCCT angiography shows an ultra-high-definition (source dataset; matrix 1024 × 1024; slice thickness/increment 0.2/0.1 mm; voxel 100 microns; convolution kernel B60f; radiation dose comparable to equivalent CT angiography of the carotids with comparable Dual-source CT of the 3rd generation) carotid bifurcation with severe mixed atherosclerotic plaque ((**A**): longitudinal view; (**B**): axial view). To be noted is the very sharp delineation of the bulky and massive calcification of the plaque both with respect to the remaining noncalcified component and with respect to the lumen definition.

Figure 4, Figure 5, Figure 6 and Figure 7 show examples of PCCT Angiography of carotid artery bifurcation.

It is important to underline that the composition analysis is not restricted to only the atomic composition, but there is some evidence that PCCT can be further used for a better assessment in the tissue composition, as demonstrated in targets other than neurovascular fields: for instance, trabecular bone microstructure analysis [50], detection and characterization of renal stones [18,51], breast tissue characterization [52], and detection, differentiation, and quantification of crystal deposits in gout [53].

### 6.5. Calcium Decomposition Algorithms

The detection and characterization of the tissues is an important step in the diagnostic process, particularly in neuroimaging. Material decomposition has mostly been studied in contrast-enhanced CT but can also be conducted in noncontrast brain CT, for instance, to discriminate between hemorrhage and calcifications. This distinction is clinically important, for example, to differentiate benign cerebral calcifications from pathologies that bear risk of bleeding (e.g., cavernous hemangiomas) or to distinguish cerebral tumors, such as oligodendrogliomas which are commonly calcified from glioblastomas which commonly contain intratumoral hemorrhages, radically changing the treatment strategy [54]. DECT has already been shown to be of value in this regard [55,56,57].

### 6.6. Dose Reduction

The attenuation values of the VNC images have been shown to be similar to true unenhanced images in all tissues; hence, VNC images may eliminate the need for true noncontrast scans in multiphasic studies, thus reducing the radiation dose [15,20]. Radiation dose savings of up to 40% have been shown by eliminating the true noncontrast phase [6].

A study by Sawall et al. found that the iodine contrast improvement obtainable with PCCT can be used to either reduce the patient dose or the amount of contrast administered to the patient [34].

The use of VNC is controversial in the literature: some authors reported significant differences in objective and subjective image quality compared to the reference, especially in the large vessels where the very high iodine concentration significantly increases the attenuation in the corresponding VNC images. Therefore, late arterial or portal-venous phase images are more appropriate for these reconstructions in multiphase studies than the arterial phase images [58,59].

Moreover, the higher spatial resolution intrinsic to the PCCT can decrease the radiation dose by 20–30% without sacrificing image quality and even up to 67–83% through an additional tin filter [17], a feature with implications in the pediatric population.

### 6.7. Targeted Contrast-Material Based Analysis

PCDs resolve the energy of each photon of the transmitted spectrum, quantify them, and classify them into energy bins and, in the presence of one or more exogenous materials with high atomic numbers, PCCT produces quantitative maps of the distribution of an individual element. The most-used exogenous material is the contrast material and, from a conceptual point of view, PCDs enable the use of new classes of contrast materials (for instance, nanoparticles, tungsten contrast media, and more) with different atomic numbers to study the biological process and/or specific accumulation sites.

In the neurovascular field, the potential applications are groundbreaking due to the necessity to track the pathological pathways at deeper levels. A recent study used gold nanoparticles to study, in an animal model, the macrophage accumulation in the vulnerable atherosclerotic plaques. This could be applied to carotid and intracranial arteries to explore and eventually detect the inflammatory atherosclerotic process, and a correlation of 0.82 was observed between the gold concentration measured within the wall and the macrophage area in 35 plaques [35,60].

Another study demonstrated in atherosclerotic carotid arteries that PCCT using the multienergy bin option combined with tungsten contrast medium had the potential to improve vessel lumen and vessel wall visualization in comparison to PCCT using iodine contrast media [36].

A similar process can also be used for the brain imaging, with specific contrast materials designed for neuro-oncologic applications, with the limit that these new contrasts should be able to move within the blood–brain–barrier (BBB) [61]. Targeted accumulation of nanoparticles by passive targeting for CT imaging has been demonstrated in numerous studies so far, especially for tumor imaging. Findings from a study on an animal model by Smilowitz et al. suggest that it may be feasible for AuNP to accumulate in intracerebral tumor masses by injecting the nanoparticles intravenously [62].

The use of new contrast materials and the potentiality of PCCT to identify different kinds of atoms open the opportunity to use multicontrast administration, delivered at the same time, thereby gaining information derived from multiple targets [37]. Cuccione et al., in a study centered on cell therapies for the treatment of ischemic stroke in an animal model, found that multicolor spectral PCCT allowed monitoring and quantification of therapeutic cells and their encapsulating scaffold transplanted in the damaged rat brain [38].

Moreover, these new contrast materials through functionalization of the nanoparticle surface with targeting moieties, such as antibodies, can actively target specific sites, such as precise surface markers of cancerous cells. There has already been application of PCCT in this field in vitro, with quantification of monoclonal antibody-conjugated gold nanoparticles targeted to lymphoma and breast cancer, aimed to detect tumor heterogeneity and to adjust the cancer treatment accordingly [63].

Peptides can also be conjugated onto the nanoparticles’ surfaces to promote their accumulation in the brain. A paper by Bao et al. conjugated a peptide to facilitate the BBB crossing of cerium oxide nanoparticle (CeONP) [64]. The authors proved that CeONP helped treat and protect the brain against stroke induced by ischemic injury after surpassing BBB. Although CT imaging was not performed in this work, the accumulation was such that the detection by PCCT could be made, indicating the potential role of CeONP as a theranostic agent for brain disorders.

## 7. Conclusions

Due to its benefits, such as increased spatial resolution, significant noise reduction, dose efficiency, reduced radiation exposure, and the optimization of the use of contrast agents and material decomposition, PCCT can improve the diagnostic value of CT in the neurovascular field.

## Figures and Tables

**Table 1:** Advantages of PCCT.

Photon-Counting Detector Property	Effect on Images
Direct conversion of X-ray to signal that is dependent on photon energy	Increased iodine signal Ability to obtain multienergy images
Smaller detector pixel size and lack of reflective septa	Improved spatial resolution Reduced radiation dose
Energy thresholds	Only quantum noise is present Reduced radiation dose Reduction of metal and blooming artifacts

**Table 2:** Studies regarding PCCT in neurovascular imaging.

Authors	Year	Model	Number of Patients	Clinical Application	Results
Symons et al. [19]	2018	In vitro In vivo (human)	16	Iodine-only images	In vitro, excellent correlation (R^2^ = 0.998) was registered between actual and calculated iodine concentrations. In vivo, PCD image noise was 9.1% lower than EID image noise. In the spectral data analysis, iodine maps had 20.7% higher CNR than nonspectral PCD.
Riederer et al. [26]	2019	In vitro Ex vivo (bovine brain)		Iodine-only images	Iodine density maps in the qualitative analysis enabled clear differentiation between blood and iodine both in vitro and ex vivo. In the quantitative analysis, measurements of the different iodine concentrations matched well with those of actual known concentrations (RMSE = 0.19).
Van Hedent et al. [27]	2018	In vitro		Iodine-only images	Conventional image attenuation was not significantly different among all samples containing blood, while virtual noncontrast attenuation showed a significant decrease with a decreasing blood component (*p* < 0.01) in all blood–iodine mixtures. Relative virtual noncontrast values were significantly different among all investigated categories (*p* < 0.01) and hemorrhagic component size was correctly estimated for all categories within a 95% confidence interval.
Michael et al. [28]	2022	In vivo (human)	37	Iodine-only images	The most favorable contrast-to-noise-ratio and signal-to-noise-ratio were detected in the PER and low keV MER. In the qualitative image analysis, the PER was superior to the MER in all rated criteria. For MER, 60–65 keV was rated as best image quality.
Schmitt et al. [29]	2023	In vitro		Artifact reduction	With metal artifact reduction, both qualitative and quantitative analysis achieved statistical difference (*p* < 0.001), demonstrating a lower degree of coil-related artifacts for PCCT in comparison with EID-CT.
Do et al. [30]	2020	In vitro Ex vivo		Artifact reduction	In vitro, EID-CT had better CNRs, less noise than HTI in Macro and Chess mode (and Macro having less noise than Chess mode). In the cadaver images, PCCT Macro-HTI showed fewer artifacts compared to EID-CT for cortical bone and bone marrow (18.3% vs. 27.6% and 35.3 vs. 60.6%, respectively). In the muscle compartment, PCD-CT Macro-HTI showed fewer artifacts than EID-CT when corrected for noise. For bone marrow, Chess-HTI performed better in terms of artifacts but worse in terms of image noise and CNR than Macro-HTI.
Lee et al. [31]	2021	In vitro		Artifact reduction	Quantitative assessment showed that the 40-keV VMI (CNR = 20.6 ± 1.2) with NMAR and MD significantly increased the contrast of the iodine-enhanced region compared with that of the conventional images (CNR = 10.4 ± 0.5) having 30 to 140 keV energy levels.
Pourmorteza et al. [32]	2017	Ex vivo In vivo (human)	21	Increased resolution	Ex vivo, PCCT images showed 8.5 ± 4.8% less image noise than EID images. In vivo, PCD image-quality scores were significantly better for GM–WM differentiation and image noise (both *p* < 0.001). In the quantitative image analysis, PCCT performed better than EID-CT for image noise, SNR of GM, WM and lateral ventricle CSF, and GM–WM contrast. In the spectral analysis, GM–WM contrast was better for the low-energy bin rather than the high-energy bin, whereas the image noise was higher in each bin than in PCD images containing all detected photons.
Dahal et al. [33]	2023	Ex vivo (human)	20	Tissue analysis	FC thickness and FC area did not show significant differences between the SPCCT-derived radiological measurements and the histopathological measurements (*p*-value range 0.15–0.51 for FC thickness and 0.053–0.30 for FC area).
Sawall et al. [34]	2020	In vitro			CNR improvement increases with increasing tube voltage for all patient sizes. The iodine contrast improvement can be used to reduce patient dose or to reduce the amount of contrast agent that is administered.
Rajendran et al. [17]	2020	In vitro Ex vivo In vivo (human)	30	Dose reduction	The PCD-CT images from the head phantom and the cadaver scans reduced the dose by 67% and 83%, for sinus and temporal bone exams, respectively, compared to EID-CT. In the sinus cohort, PCD-CT demonstrated a mean dose reduction of 67%.
Si-Mohamed et al. [35]	2021	In vivo (rabbits)		Targeted contrast-material based analysis	A good correlation was observed between the gold concentration measured within the wall and the macrophage area in 35 plaques, five per each of the 7 rabbits with atherosclerosis (r = 0.82), higher than the one observed on conventional CT images (r = 0.41).
Sartoretti et al. [36]	2020	Ex vivo (carotid artery specimen)		Targeted contrast-material based analysis	Image quality on VNCa images was significantly higher for tungsten at lower dose (*p* < 0.05). Noise was significantly lower at both dose levels for tungsten VNCa images as compared to iodine images (*p* < 0.01). Simulations indicated improved material-decomposition efficiency for tungsten than for iodine pronounced at smaller object diameters.
Cormode et al. [37]	2017	In vitro In vivo (rabbits)		Targeted contrast-material based analysis	Accurate differentiation and quantification between gold and iodine contrast media was demonstrated both in vitro and in vivo in different organs.
Cuccione et al. [38]	2020	In vivo (rats)		Targeted contrast-material based analysis	The gold labeled transplanted cells were monitored for up to 2 weeks post-intralesional injection and differentiation between the labeled cells and their embedding scaffold was also conducted successfully.

## Data Availability

Not applicable.
